# A Modern View of Tetanus

**Published:** 1918-01

**Authors:** 


***A Modern View of Tetanus.*** By Fred RANSOM, Member of the War Office Committee on Tetanus. Abs. from the *Lancet*, Dec. 22, 1917.

R. describes the action of tetanus toxin and antitoxin upon the nerves. He gives special attention to local tetanus. R., in collaboration with H. H. Meyer, has succeeded by experimental work in explaining the apparent contradiction between the fact that tetanus is due exclusively to the action of the toxin upon the central nervous system, and the appearance observed in local tetanus, when only certain areas — in animals, those near the point of injection — are affected by the tetanic spasms.

By experiments, R. and M. observed that toxin, injected subcutaneously, passes from the lymph spaces into the lymph vessels and then into the blood stream. No much is taken up directly by the blood stream from the seat of injection. In about 24 hours lymph and blood contain about the same amount of the toxin. Similarly, if the toxin is injected intravenously, in about 24 hours it finds its way into the thoracic lymph. In neither case, however, does any of the toxin find its way into the cerebro-spinal fluid, even after large doses have been administered. If the toxin is administered by lumbar puncture; in the subarachnoid space, but without injury to the pia or cord, it passes into the general blood stream and after the usual incubation period, generalized tetanus ensues. If, however, the toxin is injected intravenously, and immediately afterwards the spinal cord is injured by the injection of a drop of normal salt solution, the general tetanus is preceded by local tetanus in the muscles corresponding to the injured segment of the cord. If the injection of the toxin is made directly into the cord, a local tetanus corresponding to the injured cells ensues, and the incubation period is greatly shortened. If an animal is given a large protective dose of antitoxin, so that no free toxin can exist in the blood, and then a small dose of toxin is injected into a motor nerve, a local tetanus corresponding to the distribution of the injected nerve results. It was found, however, that the injection of toxin into a purely sensory nerve does not cause either local or general tetanus.

R. and M. conclude that tetanus toxin is conveyed to the central nervous system along the motor nerves, and not through the blood or the lymph ; and that therefore " the only route by which cells can be attacked is *via* the motor nerves ". Hence the motor nerves distributed in the infected area receive the toxin first, producing local tetanus; whereas, general tetanus must be brought on by the slower process of a general distribution.

It was found also that by injecting antitoxin into the sciatic nerves, the hind-limbs could be protected from tetanus caused by a large injection of toxin which affected all other parts of the body.

Further it was observed that by dividing the spinal cord at the 2-3 lumbar vertebrae, the front of the body could be protected against tetanus produced in the hind parts by an injection of toxin in the sciatic nerves. A control animal with undivided cord developed general tetanus from the same injection, although both animals had been given a large dose of antitoxin as soon as the tetanus symptoms appeared.

Although B. believes that the sensory nerves do not convey the toxin to the central nervous system, he finds that the sensory nerves are affected if the toxin is brought into immediate contact with them. If toxin is injected into a posterior root, hyperesthesia in the corresponding region is the result. This hyperesthesia, which he calls *tetanus dolorosus*, does not occur with motor tetanus, and therefore R. concludes that the transporting medium of the tetanus virus is not the lymph, the blood, or the sensory nerves, but the protoplasma of the motor neurons.

The author's theory of the progress of tetanus is as follows : " The toxin traveling along the motor nerves reaches the corresponding motor cells and sets up there a certain over-excitability, so that the usual normal reflex impulses which maintain the normal muscular tone become more effective, the tone of the muscles concerned is increased, they acquire a more or less distinct rigidity (stiffness), and freedom of movement is diminished ". This in animals is the condition resulting from small doses of toxin. As doses are increased, however, the receptor cells in the reflex arch are affected from the motor cells, and hence the more serious effects of the toxin contracture, and actual shortening of the muscles. Certain of the early symptoms in man, R. believes, are due to " the specific sensibility of certain central cells causing localized muscular affection ".

Localized tetanus in man is practically a new form, and is the result of the preventive use of antitoxin. Thus only a small amount of free toxin is possible in the blood and lymph, and only such toxin as is introduced by the motor nerves produces an immediate effect.

B. insists, however, that antitoxin produces its effects only in the lymph and blood. He declares that " toxin in nerves and nerve cells is inaccessible to antitoxin ", and that the latter " is not taken up by the nerves, and there is no reason for thinking that it ever gets into the nerve cells. It does not pass in any large proportion into the cerebro-spinal fluid after intravenous injection ". R., however, thinks that the therapeutic use of serum is of the highest importance, as it may neutralize some of the free toxin in the blood and lymph and prevent its ultimately entering the nervous system and causing death, when the toxin already admitted through the motor nerves is not sufficient to do so.

